# 6-Shogaol Protects against Oxidized LDL-Induced Endothelial Injruries by Inhibiting Oxidized LDL-Evoked LOX-1 Signaling

**DOI:** 10.1155/2013/503521

**Published:** 2013-02-19

**Authors:** Yun kai Wang, Ya Jun Hong, Yong hua Yao, Xiao Min Huang, Xue Bo Liu, Chun Yu Zhang, Lei Zhang, Xiaoliang Leon Xu

**Affiliations:** ^1^Department of Cardiology, Shanghai East Hospital, Tongji University School of Medicine, 150 Jimo Road, Shanghai 200120, China; ^2^Department of Oncology, Shanghai Shidong Hospital, Shanghai 200438, China; ^3^Department of Emergency, The First Affiliated Hospital of Zhejiang Chinese Medical University, Hangzhou 310006, China; ^4^Shanghai Institute of Cardiovascular Diseases, Zhongshan Hospital, Fudan University, Shanghai 200032, China; ^5^Department of Pathology, Sloan-Kettering Institute, Memorial Sloan-Kettering Cancer Center, New York, NY 10065, USA

## Abstract

Endothelial dysfunction and oxLDL are believed to be early and critical events in atherogenesis. 6-Shogaol is the major bioactive compound present in *Zingiber officinale* and possesses the anti-atherosclerotic effect. However, the mechanisms remain poorly understood. The goal of this study was to investigate the effects of 6-shogaol on oxLDL-induced Human umbilical vein endothelial cells (HUVECs) injuries and its possible molecular mechanisms. Hence, we studied the effects of 6-shogaol on cell apoptosis, cellular reactive oxygen species (ROS), NF-**κ**B activation, Bcl-2 expression, and caspase -3, -8, -9 activities. In addition, E-selectin, MCP-1, and ICAM-1 were determined by ELISA. Our study show that oxLDL increased LOX-1 expression, ROS levels, NF-**κ**B, caspases-9 and -3 activation and decreased Bcl-2 expression in HUVECs. These alterations were attenuated by 6-shogaol. Cotreatment with 6-shogaol and siRNA of LOX-1 synergistically reduced oxLDL-induced caspases -9, -3 activities and cell apoptosis. Overexpression of LOX-1 attenuated the protection by 6-shogaol and suppressed the effects of 6-shogaol on oxLDL-induced oxidative stress. In addition, oxLDL enhanced the activation of NF-**κ**B and expression of adhesion molecules. Pretreatment with 6-shogaol, however, exerted significant cytoprotective effects in all events. Our data indicate that 6-shogaol might be a potential natural antiapoptotic agent for the treatment of atherosclerosis.

## 1. Introduction

Endothelial cells (ECs) are key cellular components of blood vessels, functioning as selectively permeable barriers between blood and tissues. Under pathological conditions, endothelial cell (EC) apoptosis leads to excess neointima formation [1, 2], a lipid transport disorder [3, 4], and plaque rupture [5]. Thus, maintaining endothelial cell viability by inhibiting the induction of apoptosis could be used in the prevention and/or treatment of atherosclerosis [6, 7].

Oxidatively modiflied LDL (oxLDL) has been implicated in the development of atherosclerosis and plaque rupture by promoting lipid accumulation, proinflammatory responses, release of metalloproteinases, and apoptotic cell death of Ecs [8, 9]. oxLDL also increases endothelial expression of adhesion molecules, which recruit inflammatory cells that adhere to and migrate through the endothelial barrier. These processes are followed by endothelial dysfunction and loss of expression of antiapoptotic proteins, which in turn causes ECs to become apoptotic [10–12]. Lectin-like oxLDL receptor-1 (LOX-1) is considered the major receptor for oxLDL in human and various animal vascular endothelial cells (ECs) [13].

6-shogaol is the major bioactive compound present in *Zingiber officinale* which possesses antitumor [14], antioxidant [15], anti-inflammatory [16–18], antiplatelet aggregation [19], antihypertensive [20, 21], and antiatherosclerosis [22, 23] effects. The mechanism of anti-AS action of ginger extract is associated with a significant reduction in plasma and LDL cholesterol levels and a significant reduction in the LDL basal oxidative state, as well as their susceptibility to oxidation and aggregation [22, 23]. However, the mechanisms underlying its cardiovascular effects remain poorly understood.

The present study evaluated effects of 6-shogaol on oxLDL-induced insults to HUVECs and its possible molecular mechanisms.

## 2. Material and Methods

### 2.1. Reagents

Medium 199, fetal calf serum was purchased from Gibco, USA. Lucigenin, dimethylsulfoxide (DMSO), 3-(4,5-dimethylthiazol-2-yl)2,5-diphenyltetrazolium bromide (MTT), diphenyleneiodonium (DPI), was obtained from Sigma (St. Louis, MO). 6-shogaol was purchased from National Institute for the Control of Pharmaceutical and Biological Products (Beijing, China). The identity and purity of the compound were determined using HPLC (high-performance liquid chromatography) and 2D NMR and were >99%. 

### 2.2. Preparation of oxLDL

Venous blood from healthy adult donors was obtained from the Hangzhou Blood Center. Blood was processed for LDL separation within 1 day by sequential flotation in NaBr solution containing 1 mg/mL EDTA. Cu^2+^-modified LDL (1.0 mg protein/mL) was prepared by exposure of LDL to 5 mM CuSO_4_ for 18 h at 37°C. The extent of LDL oxidation was determined by thiobarbituric acid-reactive substances (TBARS).

### 2.3. Cell Cultures

This experiment was approved by the Research Ethics Committee of Shanghai East Hospital, Tongji University School of Medicine. After receiving written consent from the parents, we obtained fresh human umbilical cords from normal full-term neonates shortly after birth and suspended them in Hanks' balanced salt solution (HBSS; GIBCO) at 4°C. Human umbilical vein endothelial cells were cultured as previously described [24]. Briefly, HUVECs were removed from human umbilical veins after collagenase type I digestion and cultured in medium 199 containing 20% fetal calf serum, penicillin (100 U/mL), streptomycin (100 U/mL), and heparin (50 U/mL), supplemented with L-glutamine (2 mM), sodium pyruvate (1 mM), and endothelial cell growth factor (b-ECGF, 5 ng/mL), at 37°C in 5% CO_2_ on 0.1% gelatin-coated culture flasks. Endothelial cells were identified by their morphology which appears “cobblestone” mosaic appearance after reaching confluence and the presence of von Willebrand factor. Passage 3–6 HUVECs were used for experiments.

6-Shogaol was dissolved in dimethyl sulfoxide (DMSO) and stored at −20°C until use. Final concentration of DMSO in culture media was 0.1%

HUVECs were randomly divided into six groups: a normal control group, a oxLDL group, four 6-shogaol groups. Cells in the control group were incubated under the normal growth conditions. The HUVECs in the oxLDL group were incubated for 24 hours with medium containing 200 *μ*g/mL oxLDL. In the 6-shogaol groups, the cells were preincubated for 2 h with different final concentrations of 6-shogaol: 1 *μ*M, 5 *μ*M, 10 *μ*M, and 30 *μ*M, followed by a 24-hour incubation with 200 *μ*g/mL ox-LDL.

THP-1, a human monocytic leukemia cell line, was obtained from ATCC (Rockville, MD) and cultured in RPMI with 10% FBS at a density of 2–5 × 10^6^ cells/mL as suggested in the product specification sheet provided by the vendor.

### 2.4. Cell Viability Measurement

Cells were seeded at density of 5 × 10^4^ cells/mL in 96-well plates and the cell viability was measured using the MTT assay. Briefly, at the indicated time points after the treatment as before, the culture supernatant was removed, and the cells were washed with PBS, incubated with MTT (5 mg/mL) in culture medium at 37°C for another 3 h. After MTT removal, the colored formosan was dissolved in 100 *μ*L of DMSO. The absorption values were measured at 490 nm using a Sunrise Remote Microplate Reader (Grodlg, Austria). The viability of HUVECs in each well was presented as percentage of control cells. 

### 2.5. Cellular Reactive Oxygen Species and SOD Activity Measurement

Levels of cellular ROS were quantified according to a previously described method [25]. Briefly, 5 × 10^5^ HUVECs were cultured in 12-well tissue culture plates overnight and then cotreated with drugs and 2′,7′-dichlorofluorescin diacetate. After drug treatment, HUVECs were harvested and suspended in ×1 PBS buffer. Relative fluorescence intensities of cells were quantified using a flow cytometer (FACS Calibur, Becton Dickinson, San Jose, CA, USA). To evaluate the role of NADPH oxidase and LOX-1 in oxLDL-induced ROS generation, we preincubated cells with the flavoprotein inhibitor DPI (5 *μ*M) and anti-LOX-1 monoclonal antibody (mAb; 40 *μ*g/mL) for 2 h before exposure to oxLDL.

SOD activity in the homogenate was determined by an enzymatic assay method using a commercial kit (Calbiochem) according to the manufacturer's instructions. Enzyme activity was converted to units per milligram of protein. 

### 2.6. NADPH Oxidase Activity Assay

Specific NADPH-dependent O_2_^•−^ production was measured by lucigenin (5 mM) chemiluminescence as previously described [26]. The cells were pretreated with various concentrations of 6-shogaol or vehicle for 60 min, after which oxLDL (200 *μ*g/mL) was added for additional 60 min. Then cells were scraped into ice-cold HBSS supplemented with 0.8 mM MgCl_2_ and 1.8 mM CaCl_2_, disrupted by rapid freezing in liquid nitrogen followed by sonication. Oxygen radical production was measured in the presence of 5 mM lucigenin, with or without NADPH (100 mM) for 10 min. The relative light units (RLU) of chemiluminescence were read in Turner TD 20/20 luminometer. The initial 1 min of enzyme activity was monitored. Within this time period the luminescence generation was linear. The data are expressed as RLU per second per microgram cell protein.

### 2.7. Apoptosis Measurement

Apoptosis was also examined by analysis of DNA fragmentation using flow cytometry [27, 28]. HUVECs were washed and double-stained by using an Annexin V-FITC apoptosis detection kit. Annexin V has a strong Ca^2+^-dependent affinity for phosphatidylserine (PS), which translocates from the internal to the external surface of the plasma membrane as a probe for detecting apoptosis. Cells that have the loss of membrane integrity will show red staining (propidium iodide, PI) throughout the nucleus and therefore will be easily distinguished between the early apoptotic cells and the late apoptotic cells or necrotic cells. Samples were incubated at room temperature for 15 min in the dark with Annexin V and PI and quantitatively analyzed by a FACS vantage SE flow cytometer.

The activities of caspase-3, caspase-8, and caspase-9 were measured according to the kit manufacturers' instructions. In brief, after 24 h treatment by different medium conditions, HUVECs of each group were lysed and removed from culture dishes, washed twice with PBS, and pelleted by centrifugation. Cell pellets were then treated for 10 minutes with iced lysis buffer supplied by the manufacturers: caspase-3, caspase-8 assay cellular activity kit, and caspase-9 assay kit (Calbiochem). Then the suspensions were centrifuged at 10000 g for 10 minutes, and the supernatants were transferred to a clear tube. To each tube, specific substrate conjugate [acetyl-Asp-Glu-Val-Asp-p-nitroaniline (Ac-DEVD- p-NA) for caspase-3, acetyl-Ile-Glu-Thr-Aspaminotrifluoromethyl coumarin (Ac-IETD-AFC) for caspase-8 and acetyl-Leu-Glu-His-Asp-p-nitroaniline (Ac-LEHD-p-NA) for caspase-9] was added and the tubes were incubated at 37°C for 2 hours. During incubation, the caspases cleaved the substrates to form p-NA or AFC. Caspase-3 and -9 activities were read in a microtiter plate reader at 405 nm. Caspase-8 activity was read in a fluorescent plate reader at 400 nm for excitation and at 505 nm for emission. Assays were performed in triplicate and three independent experiments were performed in this study. 

### 2.8. Quantitative Real-Time PCR (qRT-PCR)

mRNA from HUVECs exposed to 6-shogaol, oxLDL, or a combination of 6-shogaol and oxLDL were prepared for real-time polymerase chain reaction (PCR) analyses of LOX-1 and GAPDH mRNA. The oligonucleotides for the PCR analyses of LOX-1 and GAPDH mRNA were designed and synthesized by Invitrogen Laboratories (Palo Alto, CA,USA). The oligonucleotide sequences for these mRNA analyses were 5′-TCTTAGCATGAATTTGGAAAT-3′ and 5′-CCCAGCTAAAGGGCCCATGG-3′ for LOX-1, and GAPDH (forward: 5′-CCA-CCC ATG GCA AAT TCC ATG GCA-3′ and reverse: 5′-TCT AGA CGG CAG GTC AGG TCC ACC-3′. A real-time PCR analysis was performed using iQSYBR Green Supermix (Bio-Rad, Hercules, CA, USA) and the MyiQ Single-Color Real-Time PCR Detection System (Bio-Rad).

### 2.9. Western Blot Analyses

Cells were lysed in a modified RIPA buffer (150 mM NaCl, 10 mM Tris, pH 7.4, 1 mM EDTA, 1% Triton X-100, 1% deoxycholic acid, 1 mM PMSF, with addition of complete TM protease inhibitor cocktail). Protein concentrations were determined by a BCA assay and separated by an 8% SDSPAGE, and then transferred to a PVDF membrane (Millipore, USA). The membrane was blocked at RT for 2 h in 5% nonfat dry milk diluted with TBST (in mM: Tris-HCl 20, NaCl 150, pH 7.5, 0.1% Tween 20). The membrane was incubated overnight at 4°C with a polyclonal rabbit anti-human LOX-1 and Bcl-2 (1 : 500 dilution; Santa Cruz Biotechnology Inc. USA). After being washed in TBS-T, the membrane was incubated for 1 h with a goat anti-rabbit IgG conjugated to horseradish peroxidase (1 : 10000 dilution; Santa Cruz Biotechnology Inc. USA). At last, the levels of LOX-1 and Bcl-2 protein were determined using Amersham ECLTM western blotting detection reagents (GE Healthcare, UK). Incubation with polyclonal rabbit *β*-actin antibody (1 : 1000 dilution; Santa Cruz Biotechnology Inc., USA) was performed as the loading sample control. Relative intensities of protein bands were analyzed by Scan-gel-it software. 

### 2.10. Overexpression or Knockdown of LOX-1

pCMV6-XL5-LOX-1 plasmids, which were constructed with full-length human LOX-1 cDNA, were purchased from Origene Technologies (Rockville, MD, USA). pCMV6-XL5-LOX-1 plasmids were transfected into HUVECs usinga FuGene 6 transfection reagent (Roche Diagnostics, Mannheim, Germany) as previously described [29]. Empty vectors were transfected as the controls. After transfection for 48 hours, HUVECs were exposed to 6-shogaol and oxLDL. Then, cells were harvested for an apoptotic analysis.

For siRNA experiments, HUVECs were transfected with LOX-1 siRNA (Santa Cruz Biotechnology). Briefly, HUVECs were cultured in antibiotic-free Dulbecco's modified Eagle's medium at 37°C for 24 hours, then the siRNA duplex solution was added. 24 hours after transfection, cells were subjected to each experiment.

### 2.11. NF-*κ*B Activation

Activation of NF-*κ*B was assessed by measuring the p65 protein-DNA binding activity in nuclear extracts of HUVECs. HUVECs were grown in 10-cm Petri dishes in endothelial cell growth medium. When the cells were 70–90% confluent, the medium was changed to 15 mL of complete M199 (20% FBS) and incubated for 2 h in the absence or the presence of 6-shogaol or anti-LOX-1 monoclonal antibody (mAb; 40 *μ*g/mL), after which HUVECs were challenged with 200 *μ*g/mL ox-LDL. For comparison purposes, HUVECs were also incubated for 1 h with pyrrolidine dithiocarbamate (PDTC), a known inhibitor of NF-*κ*B activation [30, 31]. After 1 h of incubation with ox-LDL, the reaction was stopped by washing the cells with cold PBS. Nuclear extracts were prepared according to the manufacturer's instructions, and the DNA binding activity of p65 was measured by ELISA (Active Motif, Carlsbad, CA). Briefly, nuclear extracts were added to wells previously coated with DNA containing specific sequences for the binding of p65. After incubation at room temperature for 1 h, wells were washed and sequentially incubated for 1 h with a primary antibody raised against p65 and a secondary enzyme-linked antibody. The plate was developed by addition of chromogen, and the absorbance at 450 nm was recorded in a plate-reader spectrophotometer (Spectromax 190).

### 2.12. Adhesion Molecule Expression

HUVECs were grown to confluence, then pretreated with 6-shogaol for 2 h, and stimulated cells with oxLDL (200 *μ*g/mL) for 24 h. At the end of stimulation, HUVECs were harvested and incubated with FITC conjugated anti-ICAM-1, anti-E-selectin, and anti-MCP-1 (R&D Systems) for 45 min at room temperature. After the HUVECs had been washed three times, their immunofluorescence intensity was analyzed by flow cytometry using a Becton Dickinson FACScan flow cytometer (Mountain View, CA)

### 2.13. Adhesion Assay

HUVECs at 1 × 10^5^ cells/mL were cultured in 96-well flat-bottom plates (0.1 mL/well) for 1-2 days. Cells were then pretreated with the indicated concentrations of 6-shogaol for 2 h and incubated with oxLDL (200 *μ*g/mL) for 24 h. The medium was then removed, and 0.1 mL/well of THP-1 cells (prelabeled with 4 *μ*M BCECF-AM for 30 min in RPMI at a 1 × 10^6^ cell/mL density) were added in RPMI. The cells were allowed to adhere at 37°C for 1 h in a 5% CO_2_ incubator. The nonadherent cells were removed by gentle aspiration. Plates were washed three times with M199. The number of adherent cells was estimated by microscopic examination; the cells were then lysed with 0.1 mL of 0.25% Triton X-100. The fluorescence intensity was measured at 485-nm excitation and 538-nm emission using a Labsystems fluorescence microplate reader.

### 2.14. Data Analysis

Data analysis values were expressed as mean ± SD, statistical significance was determined by Student's two-tailed *t*-test or one-way ANOVA followed by Bonferroni posttests when more than two treatments were compared. Concentration-response curves were analysed by two-way ANOVA followed by Bonferroni posttests. A *P* value of less than 0.05 was regarded as significant.

## 3. Results

### 3.1. Toxicity of 6-Shogaol to HUVECs

Treatment of mouse HUVECs with 1, 5, 10, and 30 *μ*M 6-shogaol for 24, 48, and 72 hours did not affect cell viability (data not shown). Meanwhile, after exposure for 72 hours, 6-shogaol at 60 *μ*M caused a significant 27% decrease in cell viability. When the treated concentration reached 100 *μ*mol/L, administration of  *6-shogaol*  for 48 and 72 hours, respectively, decreased the viability of HUVECs by 41% and 61% (data not shown).

### 3.2. 6-Shogaol Suppresses Low-Density Lipoprotein Oxidation and Inhibits oxLDL-Induced ROS Generation in HUVECs

**Figure 1:**
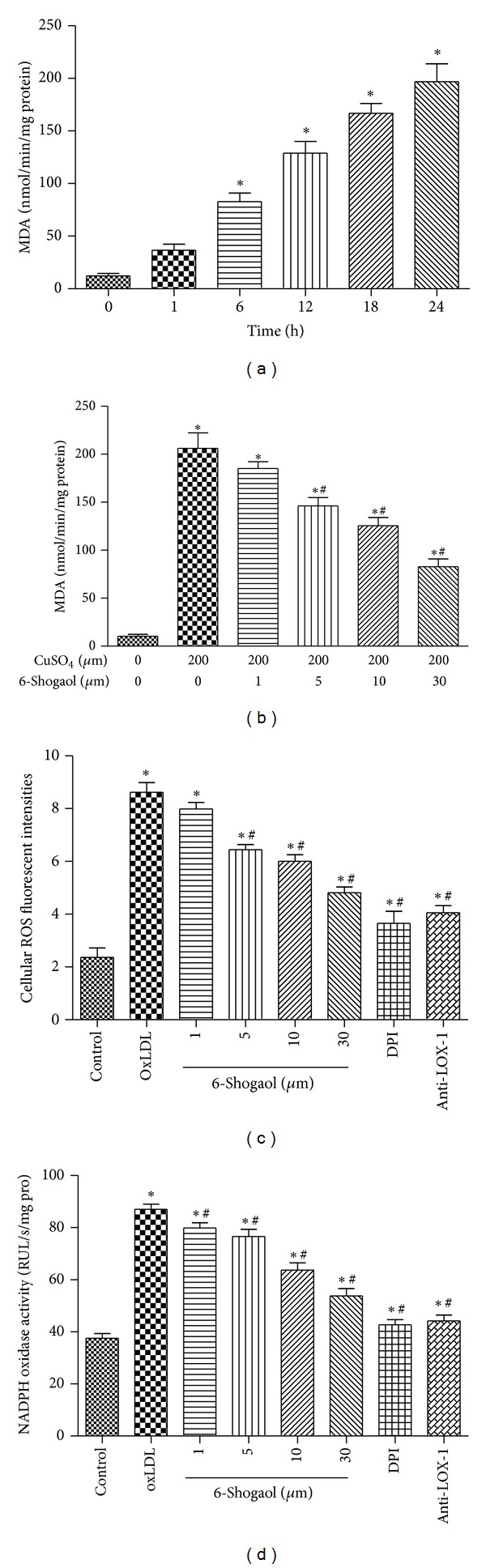
Suppressive effects of 6-shogaol on low-density lipoprotein (LDL) oxidation and cellular reactive oxygen species (ROS) production. The LDL was reacted with copper sulfate (CuSO_4_), and the amounts of malondialdehyde (MDA) were measured to evaluate the levels of oxidized (ox) LDL (a). Amounts of MDA on LDL oxidation to oxLDL were analyzed (b). Human umbilical vein endothelial cells (HUVECs) were exposed to 200 *μ*g/mL oxLDL and a combination of oxLDL and 1, 5, 10, and 30 *μ*M 6-shogaol for 24 hours. Levels of cellular ROS were quantified using flow cytometry (c). NADPH oxidative activity was determined by lucigenin chemiluminescence (d). Each value represents the mean ± s.e.m. for *n* = 6. Symbols ∗ and #, respectively, indicate that the values significantly differ from control and oxLDL-treated groups, *P* < 0.05.

Next, we observed the  *6-shogaol*  antioxidation activity by MDA measurement and used fluorescence microscopy to analyze the effect of  *6-shogaol*  on the LOX-1 mediated redox-sensitive signaling pathway in endothelial cells. As shown in Figure 1(a), when LDL was reacted with copper sulfate for 1, 6, 12, 18, and 24 hours, amounts of malondialdehyde significantly increased by 3.62-, 8.18, 12.74-, 16.47-, and 19.44-fold, respectively. 6-shogaol at 0.5 *μ*M did not affect the oxidation of LDL by copper sulfate (Figure 1(b)). Meanwhile, when the concentrations reached 1, 5, 10, 30 *μ*M, 6-shogaol decreased copper sulfate-caused oxidation of LDL by 10.3%, 29.1%, 39.1%, and 59.8%, respectively.

The intracellular ROS concentration in HUVECs was determined by measuring the intensity of DCFH fluorescence. When DCFH-DA-labeled cells were incubated in the medium for 2 h, a sudden increment in fluorescence intensity indicated the oxidation of DCFH-DA by intracellular radicals (Figure 1(c)). The production of DCFH fluorescence in HUVECs with oxLDL increased significantly to 364% of the vehicle-treated control group, whereas preincubation with 6-shogaol (1–30 *μ*M) significantly reduced the increased fluorescence induced by oxLDL in a concentration-dependent manner. In addition, oxLDL-induced ROS was abrogated by pretreatment with monoclonal antibody of LOX-1 (anti-LOX-1 mAb) or DPI (Figure 1(c)).

**Figure 2:**
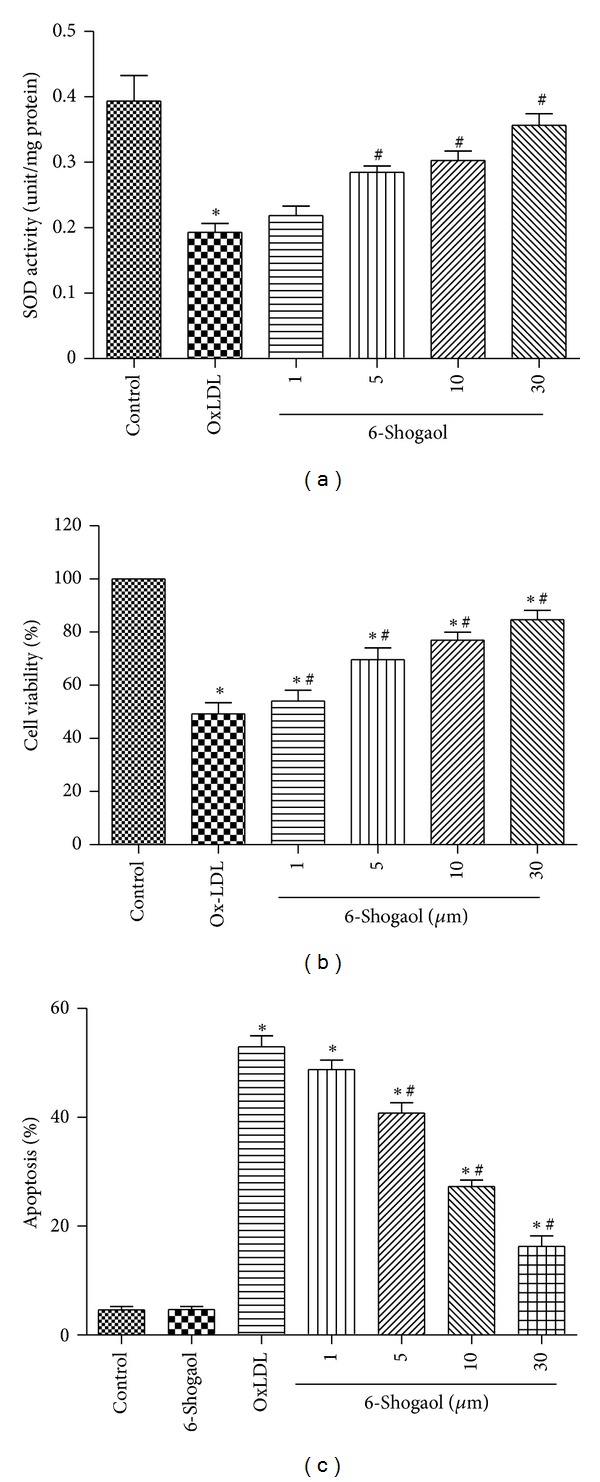
Effects of 6-shogaol against oxidized low-density lipoprotein- (oxLDL-) induced cell injuries. Human umbilical vein endothelial cells (HUVECs) were exposed to 200 *μ*g/mL oxLDL and 1, 5, 10, and 30 *μ*M 6-shogaol, and a combination of 6-shogaol and oxLDL. (a) Effects of 6-shogaol on SOD activities in endothelial cells in response to oxLDL; (b) cell viability was assayed using a trypan blue exclusion method.(c) Cell apoptosis was determined using flow cytometry. Each value represents the mean ± s.e.m. for *n* = 6. The symbols ∗ and #, respectively, indicate that values significantly differ from the control and oxLDL-treated groups, *P* < 0.05.

 ROS levels are regulated by the balance between ROS generation and antioxidant enzymes. In addition, the involved ROS is able to inactivate antioxidative enzymes that additionally increase the imbalance in favor of oxidative stress. We next turned our attention to the total activity of SOD in endothelial cells in response to oxLDL. As shown in (Figure 2(a)), 6-shogaol at 5, 10, and 30 *μ*M significantly decreased the suppression of SOD activity caused by oxLDL.

### 3.3. 6-Shogaol Reduces oxLDL-Stimulated NADPH Oxidase Activity

Endothelial NADPH oxidase is a major source of ROS in vascular endothelial cells, and atherogenic levels of LDL have been shown to induce a marked increase in NADPH oxidase-generated ROS by the endothelium. As shown in Figure 1(d), incubation of HUVECs with oxLDL (200 *μ*g/mL) for 1 h increased the NADPH oxidase activity by 132% (*P* < 0.01). 6-shogaol significantly attenuated oxLDL-stimulated NADPH oxidase activity. 6-shogaol at 10 and 30 *μ*M significantly reduced NADPH oxidase activity of HUVECs from 87.18 ± 5.06 RLU/s/mg protein to 63.92 ± 6.82 RLU/s/mg protein and 55.53 ± 3.95 RLU/s/mg protein, respectively (*P* < 0.05).

### 3.4. The Protection of 6-Shogaol against Oxidized Low-Density Lipoprotein-Induced HUVECs Injuries May Be via an Antiapoptotic Mechanism

As shown in Figure 2(b), the survival rate of HUVECs was about 52.37 ± 6.59% after exposure to 200 *μ*g/mL oxLDL. However, preincubation of HUVECs with different concentrations of 6-shogaol (1, 5, 10, 30 *μ*M) markedly increased the viability of oxLDL-treated HUVECs in a concentration-dependent manner. The treatment with 1, 5, 10, and 30 *μ*M concentrations of 6-shogaol increased the viability of HUVECs in a statistically significant fashion to 69.68 ± 4.40%, 76.91 ± 3.06%, and 83.15 ± 3.07%, respectively. In addition, no difference was seen in cell viability between cells treated with 6-shogaol (1–30 *μ*M) alone and controls (data not shown). These results suggest that 6-shogaol protected HUVECs from oxidative stress-related cellular injuries.

As shown in Figure 2(c), in the vehicle-treated control group, the percentage of apoptotic cells was 4.78% ± 1.40%. After exposure to 200 *μ*g/mL oxLDL for 24 h, the percentage of apoptosis increased to 32.43% ± 2.75%. Nonetheless, preincubation with 6-shogaol (1–30 *μ*M) for 2 h prior to ox-LDL exposure concenteration-dependently arrested the apoptosis, and the values of apoptosis were decreased to 27.93% ± 2.85%, 21.58% ± 2.27%, and 16.53% ± 2.37%%, respectively (*P* < 0.05). Moreover, the induction of apoptosis in HUVECs treated with 6-shogaol (1–30 *μ*M) alone was not observed (data not shown). 

### 3.5. The LOX-1 Receptor Participates in 6-Shogaol-Involved Cell Protection

**Figure 3:**
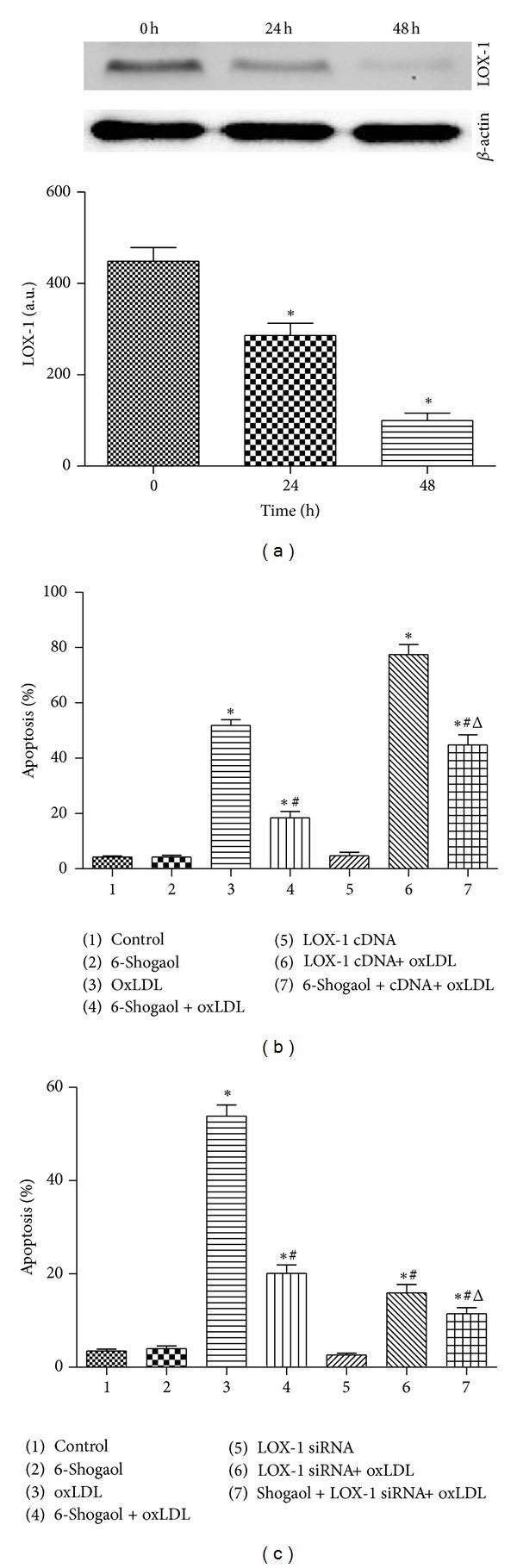
Roles of the LOX-1 receptor in 6-shogaol-involved cell protection. Human umbilical vein endothelial cells (HUVECs) were exposed to 30 *μ*M 6-shogaol, 200 *μ*g/mL oxidized low-density lipoprotein (oxLDL), and a combination of 6-shogaol and oxLDL. LOX-1 full-length cDNA was overexpressed in HUVECs or LOX-1 siRNA was applied to HUVECs. LOX-1 full-length cDNA was overexpressed in HUVECs (a), and its effects on cell apoptosis were also analyzed by flow cytometry (b). Effects of LOX-1 siRNA on cell apoptosis were determined using flow cytometry (c). Each value represents the mean ± s.e.m. for *n* = 6. The symbols ∗ and #, respectively, indicate that the values significantly differ from control and oxLDL-treated groups, *P* < 0.05. The symbol Δ means that the value significantly differs from the 6-shogaol + oxLDL-treated group, *P* < 0.05.

Application of LOX-1 siRNA into HUVECs for 24 and 48 hours decreased the levels of LOX-1 receptor (Figure 3(a)). These protein bands were quantified and analyzed (Figure 3(a)). Exposure to LOX-1 siRNA for 24 and 48 hours caused significant 32% and 79% decreases in the levels of LOX-1. Exposure of HUVECs to 6-shogaol or LOX-1 siRNA alone did not induce cell apoptosis (Figure 3(c)). The oxLDL significantly induced cell apoptosis by 59%. Treatment with 6-shogaol and LOX-1 siRNA, respectively, caused significant 58% and 65% decreases in oxLDL-induced HUVECs apoptosis. Cotreatment with 6-shogaol and LOX-1 siRNA synergistically reduced oxLDL-caused cell apoptosis by 88% (Figure 3(c)). By comparison, overexpression of LOX-1 alone in HUVECs did not affect cell apoptosis but completely attenuated  *6-shogaol*  involved protection against oxLDL-induced apoptotic insults (Figure 3(b)). 

### 3.6. Modulation of oxLDL-Induced LOX-1 Expression by 6-Shogaol

**Figure 4:**
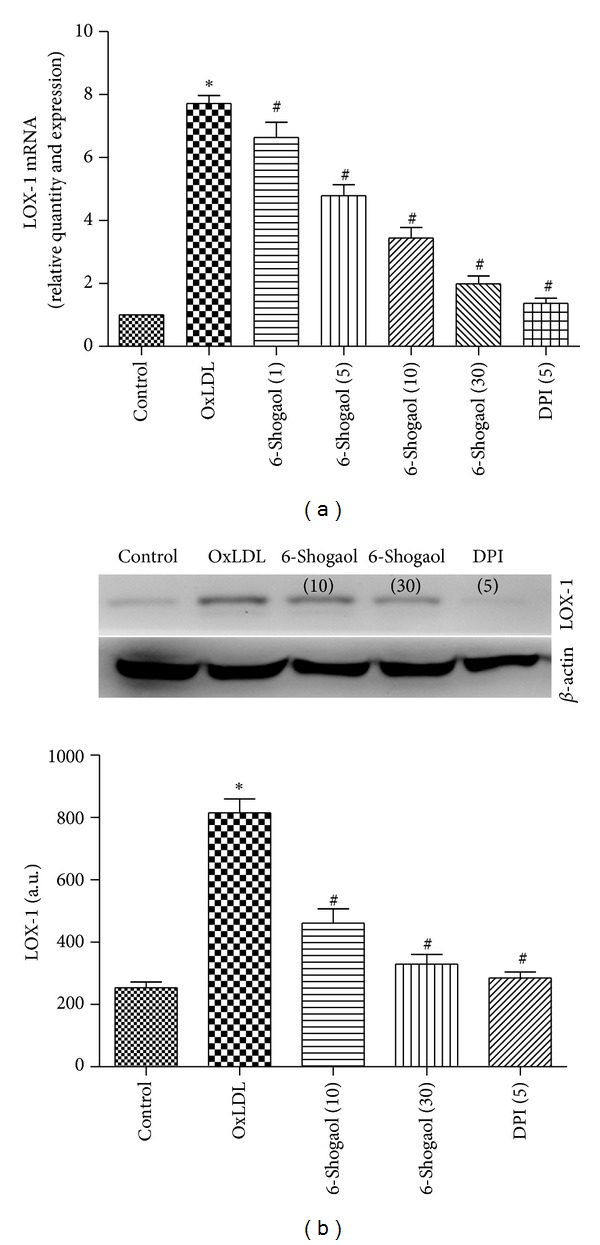
Modulation of oxLDL-induced LOX-1 expression by 6-shogaol. Human umbilical vein endothelial cells (HUVECs) were exposed to 10, 30 *μ*M 6-shogaol, 5 *μ*M DPI, 200 *μ*g/mL oxidized low-density lipoprotein (oxLDL), and a combination of 6-shogaol and oxLDL. Cellular LOX-1 mRNA (a) and protein expression were immunodetected (b).

Consistent with previous study, incubation of HUVECs with oxLDL (200 *μ*g/mL) enhanced LOX-1 expression at both the gene (Figure 4(a)) and protein levels (Figure 4(b)). Pretreatment of HUVECs with 6-shogaol for 2 h before exposure to oxLDL for 24 h resulted in suppression of LOX-1 expression in a concentration-dependent manner. Notably, pretreatment with DPI, an inhibitor of ROS production, markedly inhibited oxLDL-induced LOX-1 upregulation (Figures 4(a) and 4(b)), strongly suggesting that ROS plays a critical role in the increased expression of LOX-1.

### 3.7. 6-Shogaol Protection May Be Involved in LOX-1 Receptor-Mediated Bcl-2- Caspase Protease Pathway

**Figure 5:**
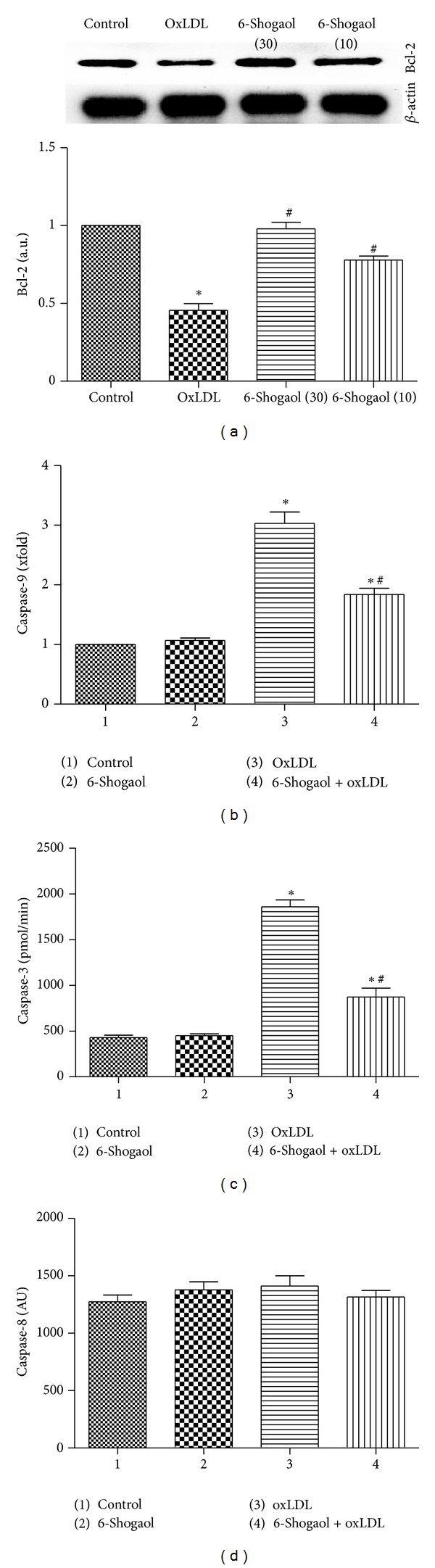
Bcl-2 expression and caspase protease activity in 6-shogaol-involved cell protection. Human umbilical vein endothelial cells (HUVECs) were exposed to 10, 30 *μ*M 6-shogaol, 200 *μ*g/mL oxidized low-density lipoprotein (oxLDL), and a combination of 6-shogaol and oxLDL. (a) Bcl-2 protein expression were quantified and analyzed. (b, c, d) the activities of caspases-9, -3, and -8 were, respectively, analyzed by fluorogenic assays using LEHD (Leu-Glu-His-Asp), DEVD (Asp-Glu-Val-Asp), and VEID (Val-Glu-Ile-Asp) as the substrate (D–F). Each value represents the mean ± s.e.m. for *n* = 6. The symbols ∗ and #, respectively, indicate that the values significantly differ from the control and oxLDL-treated groups, *P* < 0.05.

Many studies have shown that LOX-1 mediates oxLDL-induced apoptosis. oxLDL binding to LOX-1 decreased the expression of antiapoptotic proteins such as Bcl-2 and c-IAP-1, subsequently activated apoptotic signaling pathway caspase-9 and caspase-3, and finally resulted in apoptosis. Consistent with these studies, oxLDL treatment decreased the expression of antiapoptotic protein Bcl-2 (Figure 5(a)), while 6-shogaol significantly blocked the decreasing Bcl-2 expression induced by oxLDL on HUVECs. 

Caspase-3 is one of the downstream effectors of the caspase family and is involved in both the mitochondrial apoptotic pathway and the death receptor pathway. The activity of caspase-3 and caspase-9 were not affected by 6-shogaol (Figure 5). Treatment of HUVECs with 200 *μ*g/mL oxLDL led to a significant increase in activity of caspase-9 (Figure 5(b)) and caspase-3, not for caspase-8 (Figures 5(b), 5(c), and 5(d)) as compared to control; however, 6-shogaol administration significantly decreased oxLDL-induced caspase-3 and caspase-9 activation (Figures 5(b) and 5(c), *P* < 0.05).

**Figure 6:**
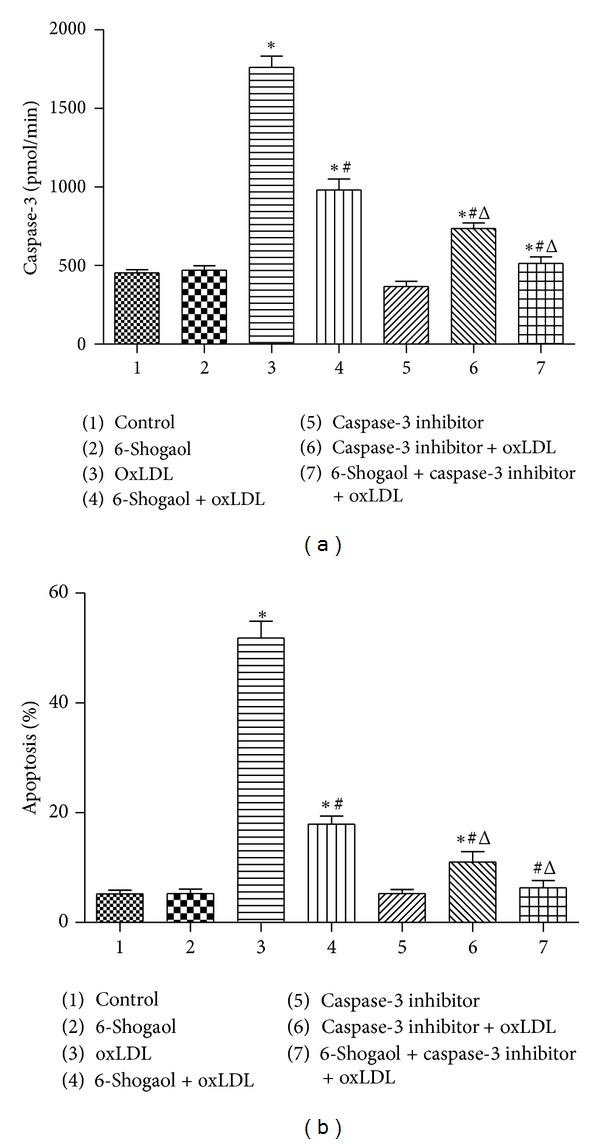
Effects of 6-shogaol, oxidized low-density lipoprotein (oxLDL), and Z-VEID-FMK on caspase-3 activity, cell apoptosis. Human umbilical vein endothelial cells (HUVECs) were pretreated with caspase-3 inhibitor DEVD-CHO (25 *μ*mol/L), for 1 hour, and then exposed to 30 *μ*M 6-Shogaol, 200 *μ*g/mL oxLDL, or a combination of 6-Shogaol and oxLDL. Caspase-3 activity was determined by a fluorogenic assay (a). Apoptotic cells were quantified using flow cytometry (b). Each value represents the mean ± s.e.m. for *n* = 6. The symbols ∗ and #, respectively, indicate that the values significantly differ from control and oxLDL-treated groups, *P* < 0.05. The symbol Δ means that the value significantly differs from the 6-shogaol + oxLDL-treated groups, ^Δ^ *P* < 0.05.

DEVD-CHO (25 *μ*mol/L), an inhibitor of caspase-3, was applied to HUVECs to further evaluate the roles of this protease in 6-shogaol-caused protection (Figure 6(a)). Treatment with DEVD-CHO significantly decreased oxLDL-induced augmentation of caspase-3 activity by 68.5% (Figure 6(a)). Cotreatment with 6-shogaol and Z-VEID-FMK completely lowered oxLDL-caused enhancement of caspase-3 activity. The oxLDL-caused HUVECs apoptosis was significantly ameliorated by 65.45% following administration of DEVD-CHO (Figure 6(b)). Simultaneous exposure to 6-shogaol and DEVD-CHO completely lowered oxLDL-induced cell apoptosis.

### 3.8. 6-Shogaol Inhibited NF-*κ*B Activation and Decreased Expression of Adhesion Molecules

**Figure 7:**
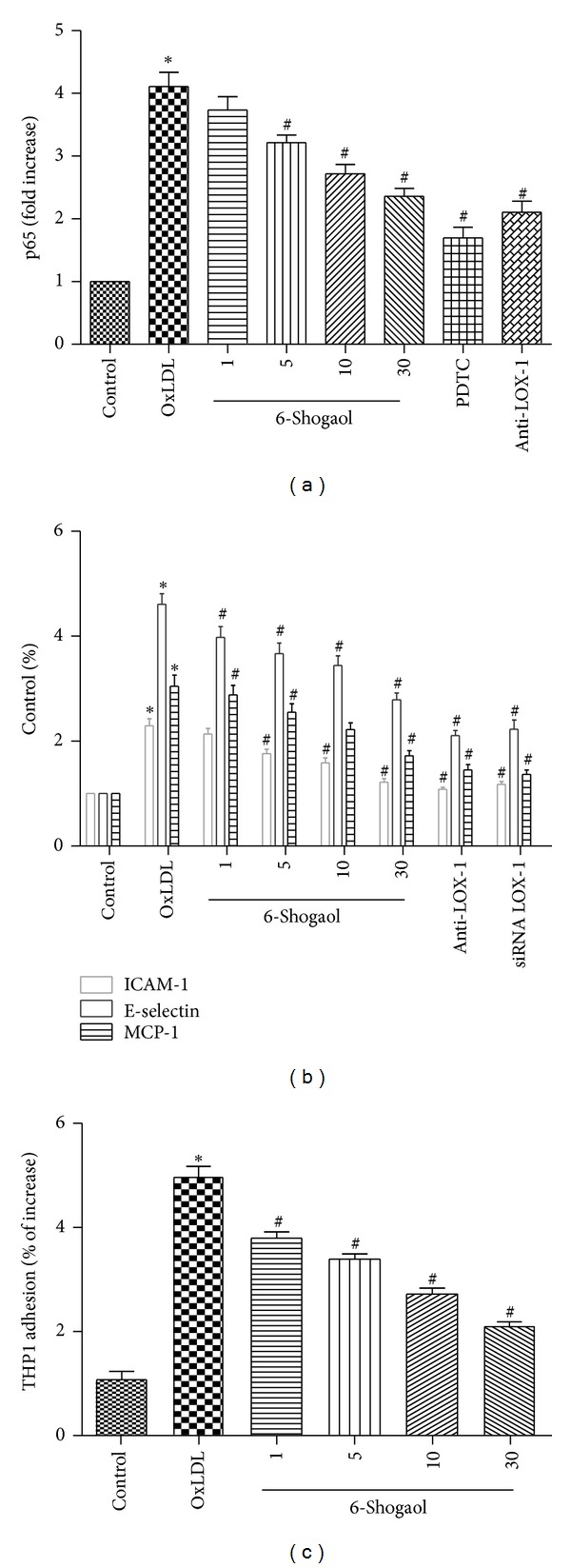
Effects of 6-shogaol on oxidized low-density lipoprotein (oxLDL)-induced adherence of THP-1 cells to HUVECs and NF-*κ*B activation and adhesion molecule expression in human umbilical vein endothelial cells. HUVECs were incubated for 2 h in the absence or presence of 6-shogaol or anti-LOX-1 monoclonal antibody (mAb; 40 *μ*g/mL), followed by the addition of 200 *μ*g/mL oxLDL. As positive control, HUVECs were also incubated for 1 h with 10 *μ*M pyrrolidine dithiocarbamate (PDTC) before incubation with oxDL. Nuclear p65 DNA binding activity was measured by ELISA. Each value represents the mean ± s.e.m. for *n* = 6. (a) Effects of 6-shogaol on NF-*κ*B activation in human umbilical vein endothelial cells. (b) Concentration-response effects of 6-shogaol on adhesion molecule expression in human umbilical vein endothelial cells. (c) Effects of 6-shogaol on oxidized low-density lipoprotein (oxLDL)-induced adherence of THP-1 cells to HUVECs. Each value represents the mean ± s.e.m. for *n* = 6. The symbols ∗ and #, respectively, indicate that the values significantly differ from the control and oxLDL-treated groups, *P* < 0.05.

OxLDL-induced ROS can activate NF-*κ*B activation, which facilitates nuclear translocation and subsequent regulation of proinflammatory gene expression [32, 33]. A shown in Figure 7(a), Activation of NF-*κ*B, as indicated by nuclear translocation and DNA binding of its p65 subunit, was decreased by 6-shogaol in a concentration dependent manner, meanwhile 10 *μ*M pyrrolidine dithiocarbamate and 40 *μ*g/mL anti-LOX-1 monoclonal antibody also exerted strong inhibition (Figure 7(a)). 

The effect of 6-shogaol on the surface expression of adhesion molecules on HUVECs exposed to oxLDL was subsequently examined. As shown in Figure 7(b), the expression levels of ICAM-1, MCP-1, and E-selectin were significantly higher in HUVECs that had been treated with oxLDL (200 *μ*g/mL) for 24 h than in the control cells (229, 304, and 460%, resp., compared with control). Flow cytometry revealed that the induction of adhesion molecule expression was significantly ameliorated by the presence of 1–30 *μ*M 6-shogaol. In addition, oxLDL-induced  *expression of adhesion molecules*  was abrogated by pretreatment with monoclonal antibody of LOX-1 (anti-LOX-1 mAb) or siRNA (Figure 7(b)).

### 3.9. 6-Shogaol Suppressed oxLDL-Induced Adherence of THP-1 Cells to HUVECs

OxLDL-enhanced recruitment, retention, and adhesiveness of human monocytes and monocytic cell lines to endothelium have been implicated in the initial stage of atherogenesis. To test the effect of 6-shogaol on monocyte adhesion to HUVECs, confluent monolayers of HUVECs were pretreated with various concentrations of 6-shogaol or anti-LOX-1 monoclonal antibody (mAb; 40 *μ*g/mL) for 2 h and then stimulated with oxLDL (200 *μ*g/mL) for 24 h, followed by incubation with THP-1 cells for 1 h at 37°C. As shown in Figure 7(c), oxLDL stimulated an increase in adherence of THP-1 cells to HUVECs (472 ± 17%, *P* < 0.05); however, the effect was significantly inhibited by 6-shogaol treatment in a concentration-dependent manner (all *P* < 0.05). 

## 4. Discussion

OxLDL is an important initiating factor for endothelial activation and injury contributing to endothelial dysfunction, one of the earliest hallmarks of atherosclerosis [8, 34, 35]; LOX-1, as the primary OxLDL receptor on endothelial cells, plays an important role in the pathogenesis of atherosclerosis [36–38]. The binding of oxLDL to LOX-1 initiates ROS formation, which in turn upregulates LOX-1 expression, thereby contributing to further ROS generation [39]. The present study shows the effectiveness of 6-shogaol, the major bioactive compound present in *Zingiber officinale*, in suppressing endothelial LOX-1 expression and LOX-1-mediated proatherogenic effects. This effect of 6-shogaol on endothelial LOX-1 expression appears to be exerted at the transcriptional level, as reflected by the parallel decrease in LOX-1 mRNA and protein levels in 6-shogaol -treated cells (Figure 3). Furthermore, pretreatment with DPI or blockade of LOX-1 with anti-LOX-1 mAb or siRNA-LOX-1 prevented oxLDL-induced ROS generation and cell apoptosis, which suggests that the binding of oxLDL to LOX-1 and the consequent formation of ROS may be the first event in LOX-1-mediated endothelial dysfunction (Figures 1 and 4). Because regulation of LOX-1 gene expression is redox sensitive [40], suppression of oxLDL-induced ROS production by 6-shogaol may contribute to the reduction of LOX-1-mediated expression of a number of proinflammatory molecules and cell apoptosis.

NADPH oxidase is recognized as the major source of ROS in endothelial cells and the increased NADPH activity has been detected in atherosclerotic arterie [37]. It has been shown that oxLDL-induced endothelial dysfunction is caused by an increase in NADPH oxidase-generated superoxide concentrations and a decrease in antioxidative enzyme activity [41], resulting in the activation of multiple ROS-sensitive signaling pathways [42]. SOD protects against superoxide-mediated cytotoxicity by catalyzing O_2_^−^ to form H_2_O_2_. This process has been shown to play a key role in atherosclerosis [43]. Consistent with the literature, our data show that 6-shogaol treatment significantly reduced the level of oxLDL-induced ROS generation (Figures 1(c) and 1(d)) and increased the level of SOD activity (Figure 2(a)). 

ROS can activate NF-*κ*B and enable nuclear translocation and subsequent regulation of proinflammatory molecules, including cytokines, chemokines, enzymes, and adhesion molecules [43]. In the present study, ROS production in HUVECs occurred within 5 min (data not shown), and NF-*κ*B was activated within 1.5 h of the addition of oxLDL. However, pretreatment with anti-LOX-1 mAb, ROS production, and NF-*κ*B activation, and ICAM-1, MCP-1, and E-selectin expression were decreased markedly, which suggests that the binding of oxLDL to LOX-1 and the consequent NF-*κ*B activation. Furthermore, our stuy showed that 6-shogaol inhibited NF-*κ*B activation (Figure 7(b)) and repressed the oxLDL-induced ICAM-1, MCP-1, and E-selectin expression (Figure 7(c)). From these, we speculated that 6-shogaol protection against oxLDL-induced endothelial dysfunction may be by blockading the binding of oxLDL to LOX-1, and subsequently decrease intracellular ROS generation and the proinflammatory molecules expression. All of these findings strongly indicate that 6-shogaol elicits antioxidative and anti-inflammatory effects.

Apoptosis, also called programmed cell death, is an important process of many pathological conditions including atherosclerosis. LOX-1 activation by oxLDL stimulates endothelial proinflammatory gene expression and production of superoxide radicals [44] and leads to activation of apoptotic signaling pathway [13, 45]. Chen et al. examined proapoptotic signaling in endothelial cells in response to oxLDL. Their findings suggested that oxLDL binding to LOX-1 subsequently decreased the expression of antiapoptotic proteins, such as Bcl-2 and c-IAP-1, then activated apoptotic signaling pathway caspase-9 and caspase-3, and finally resulted in apoptosis. Consistent with previous reports, The results presented here indicated that oxLDL induced decrease in Bcl-2 expression, but 6-shogaol completely normalized this oxLDL-induced alterations. Application of LOX-1 small interference (si)RNA into HUVECs simultaneously increased 6-shogaol protection from oxLDL-induced cell apoptosis. By comparison, overexpression of LOX-1 attenuates 6-shogaol protection. Both 6-shogaol and LOX-1 siRNA decreased oxLDL enhanced activities of caspases-9 and -3. Pretreatment with caspase-3 inhibitor DEVD-CHO (25 *μ*mol/L) synergistically promoted 6-shogaol's protection against cell apoptosis. Therefore, this study shows that 6-shogaol may protect HUVECs from oxLDL-induced apoptotic insults via downregulating LOX-1-mediated activation caspase protease pathway. 

In summary, the results from our experiments indicate that 6-shogaol prevents the oxLDL-induced LOX-1-mediated biological events in HUVECs, probably via its antioxidative and anti-inflammatory functions. Our work adds 6-shogaol to the growing list of herbal remedies whose mode of action has been at least partially revealed on a molecular level.
